# Molecular Markers of Early Immune Response in Tuberculosis: Prospects of Application in Predictive Medicine

**DOI:** 10.3390/ijms241713261

**Published:** 2023-08-26

**Authors:** Anastasiia Diatlova, Natalia Linkova, Anastasia Lavrova, Yulia Zinchenko, Dmitrii Medvedev, Alexandr Krasichkov, Victoria Polyakova, Piotr Yablonskiy

**Affiliations:** 1 St. Petersburg Research Institute of Phthisiopulmonology, Ligovskii Prospect, 2–4, 191036 St. Petersburg, Russia; 2 Biogerontology Department, St. Petersburg Institute of Bioregulation and Gerontology, Dynamo pr., 3, 197110 St. Petersburg, Russia; 3 Department of Hospital Surgery, Faculty of Medicine, St. Petersburg State University, University Embankment, 7–9, 199034 St. Petersburg, Russia; 4 Department of Radio Engineering Systems, Electrotechnical University “LETI”, Prof. Popova Street 5F, 197022 St. Petersburg, Russia

**Keywords:** tuberculosis, diagnostics, latent tuberculosis infection, immune cells, cytokines, matrix metalloproteinases

## Abstract

Tuberculosis (TB) remains an important public health problem and one of the leading causes of death. Individuals with latent tuberculosis infection (LTBI) have an increased risk of developing active TB. The problem of the diagnosis of the various stages of TB and the identification of infected patients in the early stages has not yet been solved. The existing tests (the tuberculin skin test and the interferon-gamma release assay) are useful to distinguish between active and latent infections. But these tests cannot be used to predict the development of active TB in individuals with LTBI. The purpose of this review was to analyze the extant data of the interaction of *M. tuberculosis* with immune cells and identify molecular predictive markers and markers of the early stages of TB. An analysis of more than 90 sources from the literature allowed us to determine various subpopulations of immune cells involved in the pathogenesis of TB, namely, macrophages, dendritic cells, B lymphocytes, T helper cells, cytotoxic T lymphocytes, and NK cells. The key molecular markers of the immune response to *M. tuberculosis* are cytokines (IL-1β, IL-6, IL-8, IL-10, IL-12, IL-17, IL-22b, IFNɣ, TNFa, and TGFß), matrix metalloproteinases (MMP-1, MMP-3, and MMP-9), and their inhibitors (TIMP-1, TIMP-2, TIMP-3, and TIMP-4). It is supposed that these molecules could be used as biomarkers to characterize different stages of TB infection, to evaluate the effectiveness of its treatment, and as targets of pharmacotherapy.

## 1. Introduction

Tuberculosis is one of the leading causes of death in the world. According to the World Health Organization, 1.6 million people died from tuberculosis in 2021. The incidence of tuberculosis in 2021 was 10.6 million people, including 1.2 million children [1]. Tuberculosis is common among people of all age groups. Every patient with active tuberculosis with bacterial excretion is able to infect 10–15 people, but only 5–10% of those infected show symptoms of the infection [2]. Today, about a quarter of the global population is estimated to be infected with *M. tuberculosis* [1]. Latent tuberculosis infection (LTBI) is a risk factor for the development of active tuberculosis (in about 10% of patients) [3,4], and early diagnosis is not effective enough, even with the use of modern methods.

At the present moment, to diagnose active tuberculosis the identification of the pathogen is recommended. This can be carried out using Sputum Smear Microscopy (SSM), cultural studies, and a wide range of molecular techniques, for example, nucleic acid amplification testing (NAAT) [2,5]; however, their widespread use is often curtailed owing to costs, local resources, time constraints, and operator efficiency [6]. The WHO recommends the tuberculin sensitivity test (TST) and the interferon-gamma release assay (IGRA) to diagnose *M. tuberculosis* infection [7]. At the same time, there is no gold standard for diagnosing a latent tuberculosis infection. 

*M. tuberculosis* infection is controlled by innate and adaptive immunity, but *M. tuberculosis* has protective mechanisms that suppress the immune response. According to the susceptibility to *M. tuberculosis*, there are three types of patients: resistant persons with immunogenetic mechanisms of resistance to bacteria, persons with LTBI (with a persistent immune response to tuberculosis antigens without clinical manifestations), and patients with active tuberculosis [4,8]. The most important step for preventive medicine is to identify a group of patients with LTBI who will not develop an active stage of the disease. This could be based on the identification of various correlates of the risk of tuberculosis. The following markers have been shown to be predictive of tuberculosis disease development: an upregulation of interleukin (IL)-13 and type I and II interferon (IFN)-related gene expression, elevated activation markers on T cells (e.g., expression of D-related human leukocyte antigen and a loss of CD27 expression), as well as an elevated monocyte/lymphocyte (M/L) ratio [9]. The study of the molecular and cellular aspects of the immune response in tuberculosis is necessary to explain the mechanism of the resistance, to optimize early diagnosis, and to develop targeted therapies and vaccines. In this regard, the purpose of this review was to analyze the extant data of the interaction of *M. tuberculosis* with immune cells and to identify molecular predictive markers and markers of the early stages of tuberculosis.

## 2. Pathophysiology of Tuberculosis Infection

Tuberculosis has airborne transmission: when the bacterium enters the distal respiratory tract, alveolar macrophages (AMs) phagocytize and transport it through the alveolar epithelium. Due to the inhibition of phagosome maturation at an early stage, its fusion with the lysosome and bacterium degradation do not occur. With the ESX/T7S protein and virulent mycobacterial lipids, the phagosome breaks and *M. tuberculosis* enters the cytosol. Necrotized macrophages attract monocytes, neutrophils, eosinophils, and dendritic cells (DCs) with the formation of clusters around the focus of infection; primary tuberculosis occurs. In parallel, DCs migrate to the lymph nodes, where they present antigens to naive T cells, and then they proliferate, differentiate, and migrate to the site of infection. This takes about 2–3 weeks. The accumulation of innate and adaptive immune cells leads to granuloma formation with caseous necrosis due to tissue death. With a decrease in immune protection (AIDS or aging), it is possible to reactivate the infection with a transition to active tuberculosis. Overall, 90% of patients with LTBI do not develop an active infection [10,11].

In the case of adaptive immune response dysfunction, it is possible to slow down the priming of T cells by DCs with the progression of primary infection, bacterial dissemination, and the development of active tuberculosis. Memory T cells recognize antigens and trigger cytokine cascades in active tuberculosis. This can lead to bronchopneumonia, as well as the spread of *M. tuberculosis* and the development of systemic infection [11].

The early detection of LTBI is important since these patients are at risk of developing active tuberculosis in a case of decreased immunity (HIV, cancer, immunosuppressive therapy, and aging). The concept of inflammaging implies systemic sluggish inflammation that develops during aging and is considered to be the reason for the transition from latent to active tuberculosis infection [11].

## 3. *M. tuberculosis* and Innate Immunity

When *M. tuberculosis* penetrates into the airways, the first line of protection is provided by AMs, resident macrophages of embryonic origin, and alveolar epithelial cells of types 1 and 2 (AECs). On the surfaces of the AMs and AECs, there are pattern-recognizing receptors (PPRs) that are able to bind to PAMPs (pathogen-associated molecular patterns) of foreign agents and molecular patterns associated with cell damage (DAMPs). PPRs include toll-like receptors (TLRs), Nod-like receptors (NLRs), scavenger receptors (SRs), complement system receptors, and C-type lectin receptors (CLRs) [12,13]. AMs and AECs secrete cytokines and chemokines to attract immune cells to a lesion upon contact with pathogens. The surfactant produced by AEC2 contains substances that have a direct effect on the pathogen: antimicrobial peptides, for example, LL-37 and β-defensin [14,15].

At this stage, other types of innate immune cells interact with the pathogen: neutrophils, mucosal-associated invariant T cells (MAIT), CD1-restricted lymphocytes, NKT cells, and mast cells [13,16,17]. AMs and DCs form the mononuclear phagocyte system (MPS), a class of cells that perform a specialized function of antigen presentation and lymphocyte activation [18]. Infected with *M. tuberculosis,* AMs penetrate from the airways into the interstitium, where monocyte-derived macrophages (MDMs) and interstitial macrophages (IMs) are attracted [19]. Macrophages developed from circulating monocytes actively proliferate, have a proinflammatory phenotype, have a short lifespan, and receive energy mainly due to glycolysis [20]. IMs, compared to AMs, are a stable, short-lived population [21] localized either in the alveolar interstitial tissue or the peribronchial regions [22].

During the initial stage of infection, AMs act as a niche for bacteria, where they are able to exist in the latent phase. AMs move to the lung interstitium for the dissemination of infection to MDMs and IMs [23]. IMs, on the contrary, play a protective role in tuberculosis infection [24,25], although their functional heterogeneity has been demonstrated in the lungs of mice [26]. A separate subset of lipid-rich foamy macrophages has been characterized in *M. tuberculosis*-infected lungs [27,28]. This is due to a change in the metabolic profile of infected AMs, which leads to their transformation. The exact mechanism used by *M. tuberculosis* to enhance the absorption of AM lipid droplets is currently unclear. It is known that peroxisome proliferator-activated receptor γ (PPARγ) is involved in the differentiation, inflammation, and metabolism of lipids in cells of the innate immune system, including macrophages [29], and another nuclear receptor, testicular orphan nuclear receptor 4 (TR4), is not fully clear, but it can function as a sensor of fatty acids and is expressed at a high level in macrophages [30]. *M. tuberculosis* infection activates the PPAR pathway. Both PPARy and TR4 increase the level of the macrophage receptor CD36, which absorbs exogenous lipids [28].

DCs are the most effective antigen-presenting cells and the link between innate and adaptive immunity. Immature DCs present in the mucous membrane of the lungs specialize in the absorption and processing of antigens, in particular, *M. tuberculosis*. After interacting with pathogens’ PAMPs and DAMPs, they mature, migrate to the lymph nodes, and present antigens to T lymphocytes using class I and II molecules of the main histocompatibility complex (MHC). The antigen is recognized, and the immune response is triggered. After pathogen recognition in DCs, the production of cytokines and chemokines and the expression of adhesive molecules necessary for the presentation of antigens and the stimulation of T cells increases. In most cases, fragments of the processed antigen are represented by DCs in complex with MHC-II [31,32]. 

DCs are a heterogeneous population of cells. Conventional DCs (classical, cDCs), plasmacytoid DCs (pDCs), and monocytic DCs (MoDCs) are distinguished [33]. cDCs are divided into two subtypes (cDC1 and cDC2) differing in their expression profiles of receptors and secreted cytokines. The DC subtypes exhibit different properties when infected with *M. tuberculosis*.

cDC1 is determined by the expression of CD8a/CD103 and Dec-205 in mice and CD141 in humans or by the expression of the chemokine receptor XCR1, a conservative marker for both species. cDC1 is characterized by a high ability to absorb dying cells and cross-present antigens for the immune response of cytotoxic CD8+ lymphocytes [34]. cDC2 is identified by the surface marker CD11b (in humans and mice), along with DCIR-2, CD301b, CD4, or SIRPa in mice and CD1a in humans [35]. CD103+ cDC, after infecting *M. tuberculosis,* is a functionally and phenotypically distinct subgroup of pulmonary DCs and produces mainly IL-12/23p40 heterodimers with a common p40 subunit that are necessary for the differentiation of Th1 and Th17 T helper cells. These DCs express MHC-II, CD80, and CD86 on their surfaces, as well as additional markers associated with antigen presentation and the interactions of antigen-presenting cells (APCs) and T cells: CD40, CD70, DEC-205, CD83, and PD-L2 [36,37].

PDCs have a plasma morphology and secrete a large amount of type 1 interferon (IFN-1) [38]. In response to the administration of the Bacillus Calmette–Guérin (BCG) vaccine to C57Bl/6 mice, the phagocytic activity increased in pDCs isolated from the spleen compared to CD8+ and CD8- cDC populations. At the same time, the expression of CD40, CD80, CD86, and MHC-II on cDCs increased compared to pDCs. cDCs showed higher expression of IFN-γ and IL-12p70 compared to pDCs. A higher level of TNF-α and MCP-1 expression was observed in pDCs than in cDCs. Despite the fact that pDCs actively absorb BCG, cDCs induce a more pronounced immune response, including cytokine production and antigen presentation [39]. It is likely that the function of pDCs is more related to phagocytic activity, while cDCs are responsible for the presentation of the antigen.

Inflammatory MoDCs are a subpopulation of effector cells of the lungs’ innate immunity and are activated in response to *M. tuberculosis* infection. They are identified by the expression of CD11c, CD13, Ly6C, 7/4, and TLR2 [37]. MoDCs express high levels of MHC-II, CD80, and CD86 and are predominantly located in the lung parenchyma rather than in the vasculature [40]. This suggests that MoDCs are capable of antigen presentation. Inflammatory MoDCs are multifunctional and express IL-1α, IL-1β, IL-10, TNF-α, and inducible nitric oxide synthase (iNOS) in the lungs between 2 and 3 weeks after infection with *M. tuberculosis*. Their effector functions are regulated by intracellular signaling of the type I IFN receptor [37,41]. The differentiation of monocytes in MoDCs in the presence of *M. tuberculosis* leads to a polarization shift of T helper cells from Th1 to Th2 and Th17 [42]. The functional heterogeneity of inflammatory MoDCs and their antimicrobial properties have yet to be studied in the context of understanding their role in the pathogenesis of tuberculosis.

NK cells are also involved in the response to *M. tuberculosis* infection. NK cells produce IFN-γ and transmit signals to infected DCs and AMs to promote the elimination of mycobacteria [43]. In tuberculosis, NK cells populate the granuloma and control the infectious process using various mechanisms. One of them is direct cytotoxic activity, for example, the release of perforin and granulysin by cells [44,45]. Another indirect mechanism is the transmission of signals to the adaptive immune system [46,47,48,49]. The possibility of an “innate memory” of NK cells about *M. tuberculosis* has also been described, similar to the function of memory cells in the adaptive immune system [50,51].

It can be concluded that the first line of defense against *M. tuberculosis* is provided by cells of innate immunity. The contributions of some subtypes of these cells in the pathogenesis of tuberculosis infection have been studied in sufficient detail, while others remain not fully understood. The most important function of innate immune cells is the presentation of an antigen and the activation of an adaptive immune response.

### 3.1. The Role of T Lymphocytes in the Immune Response in Tuberculosis

T lymphocytes undergo activation and an expansion of specific populations against *M. tuberculosis* antigens inside the lymph nodes. After 2–6 weeks of *M. tuberculosis* infection, the T-cell immune response can be confirmed by a delayed hypersensitivity reaction to intradermal injected tuberculin or PPD. 

When interacting with CD4+ T cells (naive T helper cells, Th0), DCs activate their differentiation into various subtypes: Treg, Th1, Th2, and Th17. In addition, differentiation is influenced by signals from TCR receptors, co-stimulating receptors, and cytokines. When cytokines bind to receptors, STAT transcription factors are activated, which are responsible for the direction of T-cell differentiation [52]. Th1 and Th17 cells are the main effector CD4+ T cells in tuberculosis. The most important proinflammatory cytokines inducing Th0 maturation in Th1 cells are IFN-γ, IL-8, and IL-12, and in Th17 cells the most important proinflammatory cytokines inducing Th0 maturation are IL-6 and TGF-β. IFN-γ and TNF-α are Th1 effectors, and their secretion is regulated by the transcription factors STAT4 and T-bet in response to intracellular pathogens. The transcription factors STAT3 and ROR-yt regulate the synthesis of Th17 effectors (IL-17A, IL-17F, and IL-22). The intracellular pathogen *M. tuberculosis* causes a Th1-mediated immune response [53].

The physiological production of IFN-γ and IL-17 is associated with the interaction between DCs and CD4+ T cells. *M. tuberculosis* can disrupt this interaction by inhibiting the binding of CD40 on DCs and the binding of its CD40L ligand on antigen-specific CD4+ T cells. The evaluation of IFN-γ production by CD4+ T cells is especially relevant in the early stages of *M. tuberculosis* infection [49]. Several studies have demonstrated a decrease in the percentage and absolute value of CD4+ cells in the peripheral blood of children and adults with active tuberculosis, which indicates their increased pool in the focus of infection [54,55].

CD8+ T cells, cytotoxic T lymphocytes in mouse lungs infected with *M. tuberculosis*, interact with MCH-I, express perforin, and lyse infected macrophages [56]. CD8+ T cells express proteins (granzymes of serine proteases), which are stored in granules and, when a lymphocyte interacts with a target cell, are transported into it through perforin. Traditionally, they are described as inducing proapoptotic pathways in target cells. It has recently been demonstrated that granzymes are involved in the degradation of the extracellular matrix and the regulation of the synthesis of proinflammatory cytokines [57]. In addition to cytotoxic T lymphocytes, granzymes are expressed in NK and NKT cells. Granzyme B is expressed in CD8+ T cells in the peripheral blood of people infected with *M. tuberculosis*, while its level is higher in patients with a latent infection compared to active tuberculosis [58]. T cells expressing granzyme A have been found in human granulomas [59]. At the same time, mice with a granzyme B knockout were no more susceptible to *M. tuberculosis* infection [60].

Human CD8+ T cells express another cytolytic protein: granulysin [61,62]. Granulysin is synthesized as a 15 kDa molecule and is cleaved to develop a form with a molecular weight of 9 kDa, which is located inside the granules in CD8+ T cells and NK cells. This protein can use perforin to penetrate a target cell. It has recently been shown that granulysin also directly forms pores and transports granzymes to target cells [63]. Granulysin is responsible for the destruction of *M. tuberculosis* inside AMs [64]. Although the mechanism of the destruction of *M. tuberculosis* with granulysin is not completely clear, it is known that the protein damages the cell wall and disrupts lipid metabolism in bacteria. Thus, granulysin, perforin, and granzymes are powerful anti-tuberculosis protection factors. Interestingly, a perforin knockout in mice did not contribute to their increased susceptibility to *M. tuberculosis* since the absence of perforin in mice leads to higher cytokine production, including IFN-γ, and compensation for the absence of the cytotoxicity of CD8+ T cells [65].

Some T lymphocytes are Foxp3-positive and perform the function of controlling the activity of other T lymphocytes. Such lymphocytes are defined as T-regulators (Treg). It is assumed that by restraining the reaction of T lymphocytes, Treg contribute to the development and preservation of the tuberculosis infection [66].

### 3.2. The Role of B Lymphocytes in the Immune Response in Tuberculosis

Although early animal studies with B-cell knockouts did not confirm their role in the pathogenesis of tuberculosis, evidence of their contribution to the body’s defense against *M. tuberculosis* is emerging. It is considered that the cellular immunity mediates the defense against intracellular pathogens, whereas the antibodies produced by B cells respond to extracellular pathogens. However, B cells produce not only antibodies but also a wide range of cytokines as competent antigen-presenting cells. This can affect the function of other immune cells, including T cells, DCs, AMs, and neutrophils, regulating their responses to pathogens [67,68].

The antigen-presenting function of B cells could promote CD4+ T-cell immunity during *M. tuberculosis* infection [69]. It has been demonstrated in the B-cell deficiency mouse model that the correct programming and inducing of *M. tuberculosis*-specified effector cells require the B cells’ antigen presentation to CD4+ T cells [70]. The level of MHC-II on the surface and the antigen-presenting activity to CD4+ T cells of lung B cells increased during *M. tuberculosis* exposure to mice with a genetic susceptibility to tuberculosis infection [71]. The antigen-presenting potential of B cells increases vaccine effectiveness, including the effectiveness of an anti-*M. tuberculosis* vaccine [72,73].

B cells are a part of the granuloma in mouse and primate models, as well as in patients infected by *M. tuberculosis.* A classic tuberculous granuloma contains a central area that consists of macrophages infected by *M. tuberculosis* and can be infiltrated by neutrophiles. It can develop into caseous necrosis or a mineralized lesion. Foam macrophages with single giant Langhans cells surround the necrosis granuloma center. An outer layer of granuloma consists of T and B cells. On the periphery of the granuloma, B cells form structures called tertiary lymphoid organs; ectopic lymphoid follicles; or, in a case presenting in the lungs, induced bronchus-associated lymphoid tissue (iBALT). These B-cell clusters are the sites of immune cell proliferation in the lungs of patients with active lung tuberculosis [68]. The effect of the ectopic lymphoid follicles formed by B cells in *M. tuberculosis* sites remains insufficiently explored. There is an opinion that the forming of B-cell follicles in the lungs of patients with active tuberculosis correlates with less infection progression [74].

Studies of B cells’ role in the pathogenesis of tuberculosis are continuing. Alterations of B-cell subsets in the peripheral blood of patients with tuberculosis have been found. In particular, it has been demonstrated that decreasing levels of naive B cells and eB5 memory B cells are necessary for long-term defense from re-infection [75,76]. 

An analysis of T follicular helper (Tfh) cells that controlled B cells’ maturation and antibody generation during various infections was conducted. During *M. tuberculosis* infection, Tfh cells accumulated in the lungs and mediated the host’s defense with proinflammatory cytokine secretion [77]. The authors described the difference between the Tfh-cell subsets: in patients with tuberculosis, Tfh1 cells were significantly decreased, whereas Tfh2 cells were increased compared to healthy donors. It is known that Tfh1 cells induce apoptosis in activated naive B cells, whereas Tfh2 and Tfh17 cells stimulate the differentiation of naive B cells in plasmatic cells and antibody production [75].

**Figure 1:**
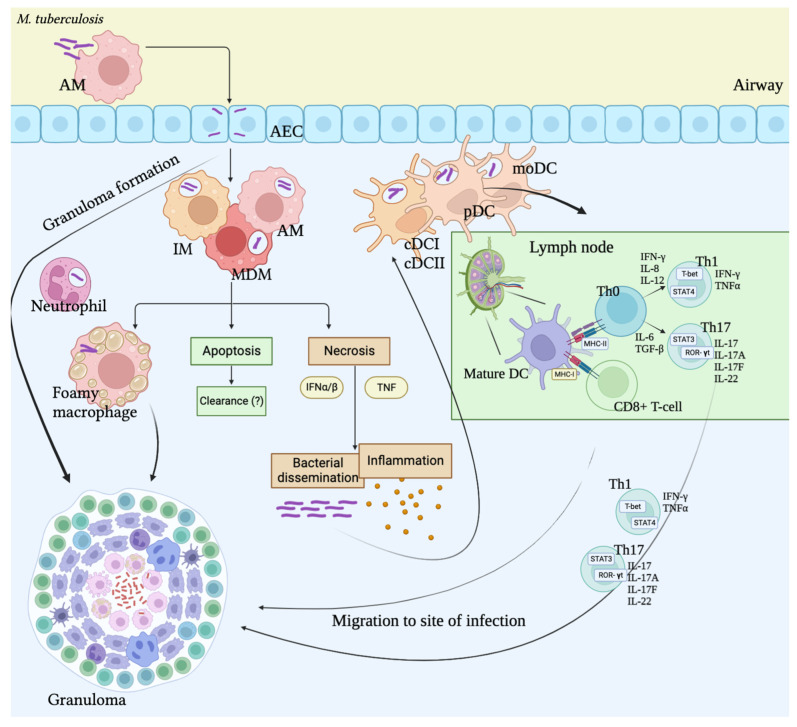
Immune response during *M. tuberculosis* infection. AMs—alveolar macrophages, IM—interstitial macrophages, MDM—monocyte-derived macrophages, AEC—alveolar epithelial cells, DCs—dendritic cells, IFN—interferon, TNF—tumor necrosis factor.

We can conclude that the pathogenesis of *M. tuberculosis* infection involves both innate and adaptive immune cells (Figure 1). Subsets of AMs and DCs present *M. tuberculosis* antigens to T cells using MHCI and MHCII. The ability of *M. tuberculosis* to use AMs as a cellular niche leads to massive cell contamination. Some infected cells enter apoptosis, while others become necrotic, which leads to bacterial dissemination, the attraction of innate immune cells, and inflammation. In lymph nodes, DCs present the antigen to T cells. Then, T cells proliferate, differentiate, and migrate to the emerging granuloma. T-killers induce the death of infected cells with perforin, granzymes, and granulysin during direct contact. T helpers are the essential source of IFN production during *M. tuberculosis* infection. B cells act not only as antibody producers but as antigen-presenting cells for T cells by releasing cytokines. Additionally, B cells form ectopic follicles that might constrain the progression of infection. It should be noted that infected cells in a granuloma are physically less available to T and B cells, which leads to difficulty in recognition and decreases cell immunity [8].

### 3.3. The Role of Immune Cells’ Cytokine Profiles in Pathogenesis of M. tuberculosis Infection

In vivo studies using animal models and clinical studies demonstrate that cytokines are significant predictors of the *M. tuberculosis* infection outcome [18]. Cytokines are involved in both early and late immune responses to *M. tuberculosis* infection. Various types of immune cells form the unique cytokine profile during the interaction with the pathogen. The imbalance in cytokine release can lead to the progression of infection. An understanding of the molecular basis of cytokine secretion during tuberculosis is important for the successful development of diagnostic and prognostic methods and pharmaceutical substances against *M. tuberculosis* [78].

Dendritic cells and macrophages. As a primary link of immune responses, AMs and DCs play an important role in recruiting cells to the infection site with the secretion of IL-1 and IL-6 [79]. Immature DCs are present in the lung mucosa and specialize to uptake and process antigens. After interactions with pathogens, DCs become mature and start to migrate in the lymph nodes, where T cells are primed by MCH and co-stimulating molecules as well as cytokine secretion [80]. According to some data, *M. tuberculosis* inhibits DC maturation, masks the presence of the pathogen, and reduces the pathogen’s ability to stimulate antigen-specific T cells. The maturation of DCs also depends on the types of receptors that recognize the antigen: in the case of the interaction of *M. tuberculosis* with TLRs, DCs are activated, characterized by a high secretion of IL-12, while the interaction with DC-SIGN prevents the activation of DCs and is characterized by a high secretion of IL-10 [81]. 

The infection of MoDCs by *M. tuberculosis* leads to the upregulation of MHC I, MHC II, CD40, CD54, CD58, and CD80 [82]. This phenotype is consistent with the activation of DCs and high production of proinflammatory cytokines (IL-12, TNF-α, IL-1, and IL-6). The cytokine profile leads to migration, processing, antigen presentation, T-cell activation, and effective cell-mediated immunity. These cells also demonstrate increases in CD83; the co-stimulating molecules CD40, CD80, and CD86; and the adhesion molecules CD58 and CD54 [83]. Other data demonstrate that DCs infected by *M. tuberculosis* produce high chemokine levels, especially the CCL3, CCL4, CXCL8, CXCL9, and chemokine receptor CCR7 levels that are necessary for NK- and T-cell migration [84].

Rv2145c is an *M. tuberculosis* protein that stimulates AMs in the lungs and spleen to synthetize IL-6, IL-10, and TNF-α by activating a mitogen-activated protein kinase (MAPK) and increasing NF-κB, TLR4, and STAT3 synthesis [85]. M1 macrophage markers during *M. tuberculosis* infection include (iNOS)/eNOS, IFN-γ, STAT-4, T-bet, SOCS3, CCR7, and CCL19/21. M1 macrophages demonstrate a lack or low expression of arginaze-1/2, CD206, CD163, MerTK, STAT-3/6, Ym1/2, Fizz1, and MRC1, whereas these molecules are predominantly expressed in M2 macrophages [86,87]. IL-8 could promote the regulation of the immune response during *M. tuberculosis* infection by direct contact with the bacteria. That leads to an increase in the ability of neutrophils and AMs to phagocytize *M. tuberculosis.* In addition, IL-8 activates CD3^+^, CD4^+^, and CD8^+^ T cells. Thus, IL-8 could be considered as a potential predictor of the development and prognosis of lung tuberculosis [88].

CD4+ T cells. IFN-γ is considered a key factor of CD4+ Th-mediated immunity (the mechanism was described above). However, a less investigated IFN-γ-independent mechanism of Th-mediated immunity has been proposed. In the signal pathway, the expression of CD153, a part of the TNF family, in Th specific to *M. tuberculosis* is in reverse correlation with the bacteria in mouse granulomas and is necessary to defend the function of CD4+ T cells [89]. CD153 expression is increased in patients with LTB compared to the active phase of infection [90]. CD4+ cells are significant for controlling and constraining the *M. tuberculosis* infection.

CD8+ T cells. Cytotoxic lymphocytes function mainly through direct contact with infected cell and the synthesis of granzymes and granulysin. However, CD8+ T cells are able to release IFN-γ, IL-17, TNF, IL-10, IL-2, and TGF-β for the reinforcement of the immune response against *M. tuberculosis* [61]. The IL-2 production of CD8+ T cells is probably important for proliferation. When a lack of IFN-γ is synthetized by CD4+ T cells, CD8+ T cells increase their production of IFN-γ, but it remains insufficient to prevent active tuberculosis [91]. The co-expression of IFN-γ, granulysin, perforin, or granzymes by CD8+ T cells has been shown in blood collected from patients infected by *M. tuberculosis* [92]. 

B cells. It has been shown that B cells release a wide range of cytokines during *M. tuberculosis* infection. In a *Macaca fascicularis* model, a role of granulomatous B cells was found in IL-6, IL-17, and, to a lesser degree, IL-10 and IFN-γ production during the acute phase of tuberculosis infection [93]. It has been demonstrated that B cells in the lungs of I/St mice, which are sensitive to *M. tuberculosis,* release high levels of the proinflammatory cytokines IL-6 and IL-11 during infection by the H37Rv strain. Atypical B cells that demonstrate decreases in IL-6 production have been obtained from patients with both active and latent tuberculosis infections [76]. 

The cytokine profile of the B cells in patients with a latent tuberculosis infection consists of the pro- and anti-inflammatory cytokines IL-1β, IL-10, IL-17, IL-21, and TNF-α during stimulation by *M. tuberculosis.* IL-1β and IL-6 are crucial for establishing and maintaining T-cell immunity against *M. tuberculosis,* which is mediated by Th17. IL-6 guides the differentiation of T cells to Th1. It has been demonstrated that IL-21 plays a crucial role in T-cell-mediated immunity against *M. tuberculosis*, reinforces the priming of CD8+ T cells, and increases the accumulation of T cells in the lungs. TNF-α reinforces T-cell-mediated immunity by activating antigen presentation and cross-priming. IL-17 and TNF-α could regulate chemokine expression and modulate immune cell recruitment, including T cells, in the site of infection. Conversely, IL-10 might attenuate the Th1-mediated immunity against *M. tuberculosis* by inhibiting TNF-α, IL-12, and HLA II expression, which constrains the antigen presentation, cross-priming, and migration of Th1 to the lungs [94]. These data point to an important role of B-cell cytokines in the regulation of cell-mediated immunity during *M. tuberculosis* infection. 

### 3.4. Role of Matrix Metalloproteases and Their Tissue Inhibitors

One of the links in the pathogenesis of *M. tuberculosis* infection is the activation of matrix metalloprotease (MMP) synthesis [95,96,97,98] and decreased tissue inhibitor of metalloproteinase (TIMP) synthesis in the lungs and other tissues. This leads to tissue degradation and dysfunction [99]. In tissues infected by *M. tuberculosis,* the expression of the *MMP1, MMP3,* and *MMP9* genes is increased, whereas *TIMP2*, *TIMP3*, and *TIMP4* expression declines. *MMP1, MMP3,* and *MMP9* gene activation is stimulated by NF-ϰB and AP-1 expressed by AMs. Dexamethasone leads to a decrease in cytokine synthesis by AMs and a normalization of MMP and TIMP gene expression. Thus, TIMP-2, -3, and -4 can be informative markers of tuberculosis progression and targets for pharmacotherapy in *M. tuberculosis* infection [91].

In another study, Kumar N. et al. analyzed the TIMP-1, -3, and -4 concentrations in blood from patients with tuberculosis and the comorbidity of tuberculosis and diabetes. They demonstrated that metformin treatment leads to decreases in the TIMP-2 and -3 levels and an increase in the TIMP-1 level in the blood. In patients with this comorbidity, the concentrations of TIMP-1, -2, -3, and -4 were increased before treatment compared to healthy donors [100]. At the same time, Esmedlyaeva et al. (2020) observed increased levels of MMP-1, 8, and 9 in patients with lung tuberculoma compared to healthy donors. TIMP-1 and MMP-3 had no significant differences between patients with tuberculoma and healthy donors [96]. The temporal dynamics of MMPs and TIMPs changing in mice during *M. tuberculosis* infection were revealed: an increase in MMP-9 during the first week and increases in MMP-2 in the blood and the TIMP-1 and TIMP-2 produced by monocytes during the fourth week of infection [99].

IFN-γ activates MMP-9 secretion and decreases the TIMP-1 and TIMP-2 produced by monocytes during *M. tuberculosis* infection. Positive regulation of IFN-γ monocyte synthesis appears due to the action of IL-1β and STAT-3. In its turn, IL-1β production is activated by the p38/MAPK signaling pathway [101] and could be inhibited by β-glucan [102]. It was shown in an investigation conducted on 681 patients with lung tuberculosis that the IL-1β blood concentration can be a predictive marker of tuberculosis severity [103]. Thus, IFN-γ, IL-1β, TIMP-1, and TIMP-2 could be prognostic markers of the course of tuberculosis, and proteins involved in the regulation of their synthesis could be potential targets in the pharmacotherapy of the disease. 

**Figure 2:**
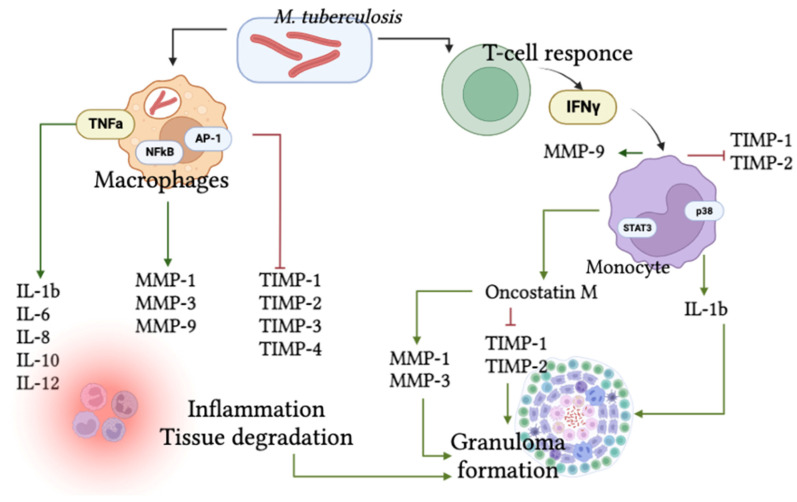
The scheme of pathological cascade involving MMPs and TIMPs during *M. tuberculosis* infection.

In addition, MAPK in lymphocytes and AMs activates oncostatin M (OSM) during *M. tuberculosis* infection. OSM interacts with TNF-α that is involved in the formation of tuberculous granulomas, stimulates MMP-1 and MMP-3 secretion, and inhibits TIMP-1 and TIMP-2 synthesis by lung fibroblasts [104]. The scheme of the pathological cascade involving MMPs and TIMPs during the course of tuberculosis is presented in Figure 2. 

## 4. Detection of *M. tuberculosis* Infection at Different Stages

The diagnosis of active tuberculosis requires the identification of the pathogen using microscopy, cultural studies, and a wide range of molecular genetics methods. The WHO recommends the tuberculin sensitivity test (TST) and the interferon-gamma release assay (IGRA) to diagnose *M. tuberculosis* infection [7]. At the same time, there is no gold standard for diagnosing a latent tuberculosis infection. 

IGRAs are based on the release of interferon-γ produced by lymphocytes sensitized with *M. tuberculosis* antigens. Their specificity is 98–100%, but they are associated with a high cost and the need for an expert laboratory. The use of the TST during a previous BCG vaccination is associated with false-positive results. The development of a test with a recombinant tuberculosis allergen and the ESAT6 and CFP10 antigens made it possible to obtain results comparable to the IGRA tests with a sensitivity of 88.7%; however, the false-positive results may be associated with allergic reactions [105,106].

Flow cytometry is a technique used to analyze the characteristics of individual cells in heterogeneous populations. Several attempts have been made to characterize the functional signature of T cells (the combination of subpopulations and their production of cytokines) associated with the stages of tuberculosis infection. For example, CD69 is a co-stimulatory receptor and a marker of early T-cell activation, and elevated levels of CD4+, CD69+, IFN-γ+ T cells are associated with early active or recent tuberculosis. The expression of CD137, a co-stimulatory molecule responsible for maintaining efficient T-cell activation, proliferation, and survival, is associated with active tuberculosis [107]. CD27, a member of the TNF-α receptor superfamily, has been found to be useful in differentiating between active and latent TB [108].

By screening 38 cytokines, Luo et al. (2019) found that the combination of CCL11, CCL22, and MCP-1 cytokines in patients’ plasma distinguishes between latent and active TB infection with a sensitivity and specificity of 87.8% and 91.8%, respectively [109]. Other plasma markers implicated as predictors of treatment outcome include IL-6, MCP-1, VEGF, heme oxygenase-1, matrix metalloproteinases, serum amyloid, IL-11 receptor antagonist, and 2-antiplasmin [110]. However, the mycobacterium-specific immune response is likely to be heterogeneous in different human populations and may depend on HIV co-infection, heredity, and some other exogenous factors, so further studies are needed to validate the above methods.

## 5. Conclusions

An analysis of the interaction mechanisms between *M. tuberculosis* and immune cells (AMs, DCs, B cells, T helpers, cytotoxic T cells, and NK cells) made it possible to identify the most significant molecular markers of these processes, which included the cytokines IL-1β, -6, -8, -10, -12, -17, -22, IFNγ, TNFα, and TGFβ; the matrix metalloproteases MMP-1, -3, and -9; and the inhibitors TIMP-1, -2, -3, and -4. 

To differentiate the early stages of tuberculosis infection and predict the course of the disease, we assume that the following markers may be more significant: the increase in IFN-γ production by CD4+ T cells; CD69 as a co-stimulatory receptor and a marker of early T-cell activation; elevated levels of CD4+, CD69+, IFN-γ+ T cells; and the expression of CD137 and CD27. As a primary link in the immune response, cytokines are significant predictors of *M. tuberculosis* infection outcomes, including AMs and DCs with the secretion of IL-1 and IL-6 as well as IL-8 as an activator of CD3+, CD4+, and CD8+ T cells—a potential predictor of the development and prognosis of infection. The IL-1β blood concentration could be a predictive marker of disease severity as a regulator of IFN-ɣ monocyte synthesis. IFN-γ, IL-1β, TIMP-1, and TIMP-2 could also be prognostic markers of the course of tuberculosis. Other plasma markers implicated as predictors of treatment outcome include IL-6, MCP-1, VEGF, heme oxygenase-1, matrix metalloproteinases, serum amyloid, IL-11 receptor antagonist, and 2-antiplasmin.

These molecules can be used for the differential diagnosis of the various stages of tuberculosis infection, the evaluation of the effectiveness of its treatment, and as targets for the pharmacotherapy of the infection.

## Data Availability

There are no new data were created.
